# Analytical validation and establishment of reference intervals for a ‘high-sensitivity’ cardiac troponin-T assay in horses

**DOI:** 10.1186/s12917-016-0737-1

**Published:** 2016-06-13

**Authors:** E. Shields, I. Seiden-Long, S. Massie, S. Passante, R. Leguillette

**Affiliations:** University of Calgary Faculty of Veterinary Medicine (UCVM), 3330 Hospital Dr. NW, Calgary, T2N 4 N1 AB Canada; Foothills Medical Centre, University of Calgary Faculty of Medicine and Calgary Lab Services (CLS), Room C618B, 1403-29th St. NW, Calgary, T2N 2 T9 AB Canada

**Keywords:** Horse, Blood chemistry, Myocardium, Muscle

## Abstract

**Background:**

Cardiac troponin-I assays have been validated in horses.’High-sensitivity’ cardiac troponin assays are now the standard in human cardiology. *Objective:* Appropriately validate the’high-sensitivity’ cardiac Troponin-T (hscTnT) assay for clinical use in horses, establish reference intervals, determine the biological variation, and demonstrate assay utility in selected clinical cases. *Methods*: Analytical validation of the Roche hscTnT assay included within- and between-run precision, linear dose response, limit of quantitation (LoQ), stability, and comparison with cTn-I (iSTAT). Reference intervals and biological variation were determined using adult, healthy, Non-Competition Horses (N = 125) and Racing-Thoroughbreds (N = 178). HscTnT levels were measured in two horses with cardiac pathology.

**Results:**

The hscTnT demonstrates acceptable within-run (L1 = 6.5 ng/L, CV 14.9 %, L2 = 10.1 ng/L, CV 8.7 %, L3 = 15.3 ng/L, CV 5.4 %) and between-run precision (L1 = 12.2 ng/L, CV 8.4 %, L2 = 57.0 ng/L, CV 8.4 %, L3 = 256.0 ng/L, CV 9.0 %). The assay was linear from 3 to 391 ng/L. The LoQ was validated at 3 ng/L. Samples demonstrated insignificant decay over freeze-thaw cycle. Comparison with cTnI assay showed excellent correlation (range: 8.0–3535.0 ng/L, R^2^ = 0.9996). Reference intervals: The upper 95^th^ and 99^th^ percentile of the hscTnT population distribution were 6.8 and 16.2 ng/L in Non-Competition Horses, and 14.0 and 23.2 ng/L in Racing-Thoroughbreds. Between-breed, diurnal effect, and between-day variation was below LoQ. Two clinical cases with presumed cardiac pathology had hscTnT levels of 220.9 ng/L and 5723.0 ng/L.

**Conclusions:**

This benchmark study is the first to comply with CLSI guidelines, thus further establishing the performance characteristics of the hscTnT assay, and reference intervals in healthy horses. Two clinical cases demonstrated further the clinical utility of the assay.

## Background

As acknowledged in a recent joint consensus statement, subtle or occult cardiac abnormalities are often difficult to diagnose in horses [1]. Similarly, determining the impact of any cardiovascular abnormality on present or future performance, on rider or driver safety, and the long-term effects on health and longevity can be problematic [1]. Early detection of cardiac pathologies would provide the opportunity to recognize occult disease and allow intervention prior to potentially more serious outcomes such as cumulative cardiac damage or sudden death.

One cardiac diagnostic tool that has been adopted for use in veterinary medicine is the troponin assay. Of the three troponin subunits, only cardiac Troponin-T (cTnT) and cardiac Troponin-I (cTnI) have unique amino acid sequences expressed as cardiac muscle-specific isoforms, and are encoded by different genes than the troponins expressed in skeletal muscle [2]. Since 2000, after an extensive search for a biomarker of myocardial damage with sufficient analytical sensitivity and specificity, troponin assays have been deemed the test of choice for detection of acute myocardial injury (AMI) in humans [3]. The assays have evolved through numerous generations, each of which has undergone improvements in analytical performance. Troponin-I and T assays with total imprecision ≤ 10 % at the 99^th^ percentile, and measurable normal values below the 99^th^ percentile in at least 50 % of healthy individuals are now classified as’high-sensitivity’ assays in humans [4]. Older generation (‘non-hs’) troponin assay use in veterinary medicine has been fairly widespread (reviewed by Rossi et. al.) [5], and recently two reports use the hscTnT assay [6, 7]. There are problems however, associated with the clinical utility and application of the troponin assays reported in the equine literature to date [6, 8–12]. These include: 1)- The general lack of appropriate analytical validation which can lead to erroneous interpretation of results and subsequent designation of reference ranges and threshold cut-off points. 2)- The large number of commercially available troponin assays that differ from one another in terms of antibody-epitope specificity and preclude direct comparison of results from various studies. 3)- The use of older generation troponin assays that lack the sensitivity to detect occult or potentially cumulative myocardial injury with the degree of certainty needed in veterinary sports medicine to explore the effects of subclinical myocardial damage. With’high-sensitivity’ (5^th^ generation) cardiac troponin assays now available, our aim was to validate a human automated’high-sensitivity’ cardiac Troponin-T (hscTnT) Roche Diagnostics assay for use in horse plasma, and to establish hscTnT reference intervals in adult, healthy, Non-Competition Horses and Racing-Thoroughbreds as presented in 2014 [13]. This validation study of the Roche hscTnT assay for use in horses provides additional valuable information by complying with the recommendations of the Clinical and Laboratory Standards Institute (CLSI), the National Academy of Clinical Biochemistry (NACB), and American Society of Veterinary Clinical Pathology (ASVCP) [14–17] for the first time.

## Methods

1. Genetic homology
  Utilizing NCBI protein BLAST, *Homo sapiens* cardiac Troponin-T amino acid residues of epitopes recognized by capture MAb (M7) (125–131:DRIERR) and detection MAb (M11.7) (136–147:EQQRIRNEREKE) for the hscTnT assay were compared to *Equus caballus* cardiac and skeletal Troponin-T (Fig. 1).

**Fig. 1:**
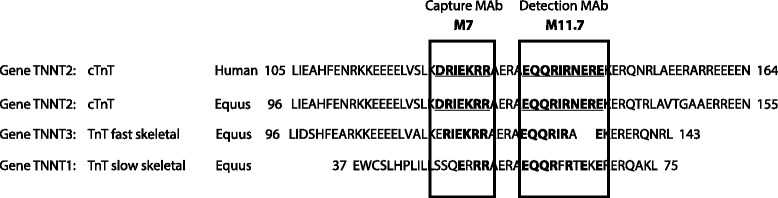
Amino acid sequence alignment of human cardiac Troponin-T (Type 2) adult isoform (cTnT) with horse (*Equus caballus*) cardiac Troponin-T (Type 2) and homology with equine fast and slow skeletal muscle TnT using NCBI protein BLAST. Amino acid sequences include the epitope regions against which the Roche, Cobas-e601 capture (M7) and detection (M11.7) antibodies are directed
2. Analytical validation of ‘high-sensitivity’ cardiac Troponin-T assay
  Roche Diagnostics cardiac Troponin-T ‘high-sensitivity’ assay, Cobas-e601 Analyzer
    HscTnT assay, a 5^th^ generation electrochemiluminescence immunoassay, was used on the Cobas-e601 Analyzer for analysis of equine plasma samples, complying with manufacturer’s instructions and CLS internal quality controls systems. The manufacturer determined the analytical characteristics for this assay in accordance with the CLSI-EP17-A2 requirements. The analyzer was calibrated using human cTnT standards supplied for use of the hscTnT assay kit with human plasma.
  Equine Troponin-T samples
    Purified equine cardiac troponin used in linearity and precision studies was obtained from harvested equine myocardium as previously described [18]. Plasma samples were collected from all live horses, by jugular venipuncture in 5 mL lithium-heparinized tubes. Specimens were centrifuged at 2000 x *g* for 10 min, separated, frozen within 90 min, and stored at−80 °C until batch analyzed. Degree of hemolysis in all samples was assessed using the serum indices instrument application on the Roche Cobas-e601 platform^2^ and no specimen included in this study reached the hemolysis threshold to be excluded.
  Tissue specificity
    Homogenates of cardiac and skeletal muscle harvested from a healthy horse were prepared as previously described [19] and subsequently diluted.^3^ Dilutions used were within the linear range of the hscTnT assay (cardiac: 100,000 fold, skeletal muscle: 10,000 fold). The tissue reactivities, expressed in ng/L wet weight, were corrected for the dilution factor and are presented as means ± SEM. Tissue Reactivity (ng/g of wet tissue) = *x* hscTnT(ng/L) x dilution factor ÷ weight of muscle sample(g) x dilution of original homogenate muscle stock.
  Linear dose response
    N = 10 dilutions were prepared from purified equine cardiac troponin stock by serial 1:2 dilution (from 1:100 to 1:0.2) using normal equine plasma (hscTnT 3.28 ng/L) as diluent. Triplicates of the resultant 11 samples (mean hscTnT levels ranging from 8.0 to 3526.7 ng/L) were analyzed to detect linearity over the expected measurable range.
  Assay precision
    Within-run precision was evaluated by assaying plasma samples from 3 horses containing 3 different previously determined (Roche Diagnostics hscTnT assay) concentrations of hscTnT (L1 = 6.5 ng/L, L2 = 10.1 ng/L, L3 = 15.3 ng/L) 10 times in the same run. Between-run precision was evaluated by assaying prepared quality control samples consisting of purified equine cardiac troponin diluted with equine plasma at 3 levels (L1 = 12.2 ng/L, L2 = 56.9 ng/L, L3 = 254.0 ng/L) over 3 months and 10 different runs.
  Functional sensitivity
    The limit of quantitation (LoQ), defined as the lowest analyte concentration that can be reproducibly measured with a between-run CV ≤ 10 %, was determined using 5 serial dilutions of a stock, diluted, purified equine cardiac troponin sample (hscTnT range 4-37 ng/L), and analysing each dilution 5 times in 1 day.
  Stability study
    Stability of samples was tested at−80 °C for a period of 24 months. Samples were measured at the start and end of the testing period and the average difference between the measured values before and after storage was calculated. Criteria for significant change: average difference of less than 2X the between-run standard deviation of the control level closest to the mean of samples measured.
  Comparison study
    Eleven diluted, purified equine cardiac troponin samples of various increasing, known hscTnT levels (previously determined using Roche Diagnostics assay), were run in duplicate on the iSTAT for equine cTnI analyte levels allowing for comparison of hscTnT to older generation cTnI results.
3. Reference intervals and biological variation of hscTnT levels in healthy horses
  Two subset populations were included in the study and sampled by convenience (summer 2013): one group of Non-Competition Horses with varying levels of routine physical activity and a second group of Racing-Thoroughbred geldings (Table 1). These populations provide reference intervals of troponin levels for application of the assay in sports medicine and exercise physiology in addition to general application in clinical cardiomyopathy cases. The study was approved by the UCVM-Veterinary Sciences Animal Care Committee and consent was obtained from the animals’ owners.

**Table 1:** Comparison of ages, sexes, breeds and resting heparin plasma hscTnT concentrations in horses

Number	125	178	1	1
	Non-Competition Horses	Racing- Thoroughbreds	Clinical Case I	Clinical Case II
Median Age (Range)	5 (2–24)	9 (4–18)	11	14
Sexes	56 Mares, 69 Geldings	178 Geldings	Gelding	Mare
Breed(s)	TB, QH, Drafts, Arabians, WB	TB	TB	QH
Resting hscTnT Mean (±SD), ng/L	3.6 (±2.1)	5.4 (±3.8)	221.0^a^	5723.0^b^
hscTnT Median, ng/L	3.0	4.0^c^	N/A	N/A
hscTnT Range, ng/L	3.0–17.0	3.0–24.0	N/A	N/A
hscTnT 95 % URL (90 % CI), ng/L	6.79 (6.0–12.0)	14.0 (10.0–18.0)	N/A	N/A
hscTnT 99 % URL (90 % CI), ng/L	16.2 (N/A)	23.2 (N/A)	N/A	N/A

Comparison of ages, sexes, breeds and resting heparin plasma hscTnT concentrations from 303 apparently healthy horses (Non-Competition Horses and Racing-Thoroughbreds) as well as two clinical cases

^a^Heparin plasma hscTnT sample taken 5 h after race-induced upper respiratory crisis event

^b^Heparin plasma hscTnT sample taken 41 h after initially presented to clinic

^c^Indicates a significant difference from Non-Competition Horses

    Non-Competition Horses: Plasma samples were taken from 125 non-performance, healthy horses (ages 2–24; median 5 years, 69 geldings, 56 mares) represented by Quarter horses, Quarter horse-crosses, Thoroughbreds, Warmbloods, Draft horses, and Arabians.
    Racing-Thoroughbreds: Plasma samples were taken from 178 healthy, fit, actively competing Thoroughbred Chuckwagon racing geldings (ages 4–18; median 9 years) when the horses were at rest in the morning prior to races.
  For both populations, a physical examination with heart auscultation was performed, and Telemetric ECG was run stall-side and observed for two minutes on all horses. Horses were excluded for abnormal physical exam findings, murmurs Grades III or greater, or any arrhythmia other than second-degree atrioventricular block. Horses had no known history of congenital or acquired cardiac abnormalities.
  Breed, diurnal and intra-individual variation
    Evaluation of differences in hscTnT levels between breed was determined by sample collection from 25 horses representing Warmbloods, Thoroughbreds, Quarter Horses, Arabians and Draft Horses (5 horses of each breed). In addition, diurnal and intra-individual variation was assessed by sample collection from each horse every morning and evening for 5 consecutive days.
4. Two selected case studies demonstrating clinical use of hscTnT assay
  Case one
    An 11 year old Thoroughbred Chuckwagon gelding passed a mandatory racetrack veterinary physical and lameness examination on the morning of a race. He had no history of previously administered medications. The gelding later presented with near-collapse event at the end of his race secondary to an acute upper airway obstruction. The horse was diagnosed stall-side, via auscultation, with ventricular tachycardia immediately following racing. Telemetric ECG was subsequently run stall-side intermittently over 48 h. Plasma hscTnT analyte levels were measured at 5 and 72 h following the event.
  Case two
    Fourteen year old Quarter Horse mare, 3 months pregnant, diagnosed with *Neorickettsia risticii* by PCR and showing acute clinical signs of cardiacimpairment, hypovolemic shock, and systemic inflammatory response syndrome (SIRS). Diagnostic testing was completed to document the status of the patient in light of the primary colitis pathology (venous hematologic, blood chemistry, venous/arterial blood gas, urinalysis, fractional excretion electrolyte ratios, and fecal PCR). Cardiac evaluation included physical examination, heart auscultation, 24-h Telemetric ECG, and plasma hscTnT analyte levels, but lacked cardiac ultrasonography (due to logistical restrictions associated with isolation hospitalization of a severe diarrhea case). Following euthanasia, post-mortem examination with selected histology was performed.

### Statistical analysis

Linearity and LoQ were determined using Analyse-it software^5^. Linearity of the assay was assessed by linear and polynomial regression of the data and difference plot with Total Error criteria. A Weighted Deming fit was used to compare the fit between the hscTnT assay versus the cTnI assay. The hscTnT population distributions were not normal using the Kolmogorov-Smirnov test. CLSI-28A3C guidelines recommend Dixon’s and Tukey’s techniques for outlier detection, however both are based on the assumption that there is a normal distribution to the population data [14]. Due to the severity of the right-skewed population in this case, normalization of the data is unreliable with transformation techniques such as Box-Cox transformation. Consequently, visual inspection was selected as a screening method and no outliers were detected in the populations tested. The Mann-Whitney *U* test was utilized to test the difference between the hscTnT levels in the two horse sub-populations. Nonparametric methods, complying with CLSI guidelines, were used for determination of the 95^th^ and 99^th^ percentiles and 90 % confidence intervals for horses from each population.

## Results

**Fig. 2:**
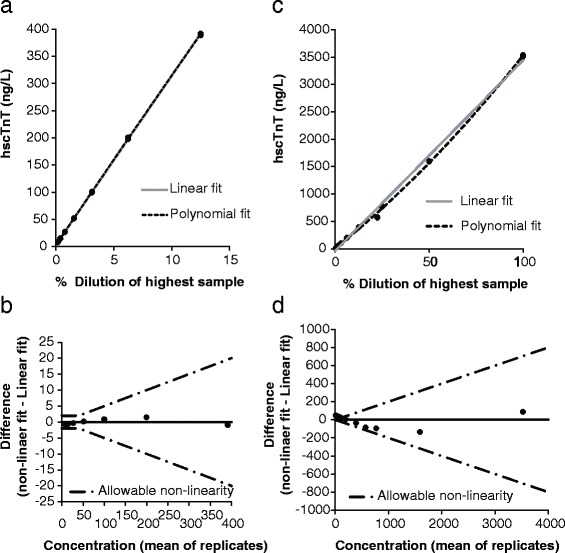
Plot of the linearity (triplicates of increasing concentrations of equine hscTnT) of the Roche, Cobas-e601 hscTnT assay. **a**, **b** Linear to a level of 391 ng/L (±2 ng/L or 5 %). **c**, **d** Linearity run up to 3500 ng/L shows over-recovery
  Assay precision
    Within-run precision was: L1 = 6.5 ng/L, CV 14.9 %, L2 = 10.1 ng/L, CV 8.7 %, L3 = 15.3 ng/L, CV 5.4 % and between-run precision was: L1 = 12.2 ng/L, CV 8.4 %, L2 = 57.0 ng/L, CV 8.4 %, L3 = 256.0 ng/L, CV 9.0 % (Table 2). In humans studies, the required precision is 10 % within-run CV at the upper limit of normal for the population. The horse specimens have < 10 % CV at the levels closest to the upper reference limit (99^th^ percentile) in both horse populations tested (Table 2).

1. Analytical validation of ‘high-sensitivity’ cardiac Troponin-T assay
  Genetic homology
    The epitopes detected by this commercial hscTnT assay were conserved between humans and horses with 100 % homology detected when comparing human adult cardiac isoform cTnT and equine cTnT [XP_005614937.1], while only 80 % [XP_005898432.1] and 56 % [XP_005896826.1] conservation was shown between human adult cTnT and equine fast and slow skeletal muscle respectively (Fig. 1).
  Tissue specificity
    Although there was some minimal detectable reactivity from equine skeletal muscle in the hscTnT immunoassay, hscTnT reactivity of cardiac muscle was 256.2 times greater than that in skeletal muscle (2.49 x10^7^ and 9.75 x10^4^ ng/g wet weight respectively).
  Linear dose response
    While linear dose response was tested in the range of 8–3527 ng/L, the assay was linear over the hscTnT range of 3–391 ng/L (±2 ng/L or 5 %). Above 391 ng/L there is bias in the regression with over-recovery of the analyte demonstrated and even when the linearity criteria are widened (to ±5 ng/L or 20 %) the response is significantly non-linear (Fig. 2).

**Table 2:** Precision of the hscTnT assay (Roche Diagnostics, Cobas-e601) for measuring equine plasma

	Level 1 (L1)	Level 2 (L2)	Level 3 (L3)
Within-Run hscTnT Mean (CV), ng/L	6.5 (14.9 %)	10.1 (8.7 %)	15.3 (5.4 %)
Between-Run hscTnT Mean (CV), ng/L	12.2 (8.4 %)	57.0 (8.4 %)	256.0 (9.0 %)

*Abbreviations*: *CV* Coefficient of variation (in %)

Precision of the hscTnT assay (Roche Diagnostics, Cobas-e601) for measuring equine plasma hscTnT of three increasing levels (Level 1, 2, 3) on 10 separate occasions
  Functional sensitivity (Limit of Quantitation)
    The LoQ was validated at 3 ng/L (using a 10 % CV).
  Stability study
    The stability of the samples was excellent and demonstrated slight, but not statistically significant decrease in hscTnT analyte over the average 24 month period between free-thaw cycles (mean = 15.8 ng/L, average difference = 1.75 ng/L).
  Comparison study
    Comparison with cTn-I assay showed excellent correlation (range: 8–3535 ng/L, R^2^ = 0.9996). Bias was evident in the Deming regression results [hscTnT = (0.6 ± 0.0028)*(cTnI*1000)–(11.5 ± 5.5)] (Fig. 3).

**Fig. 3:**
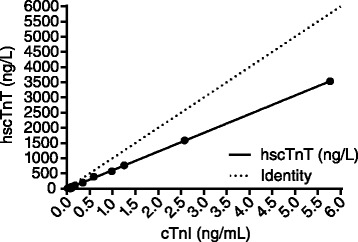
Correlation between cTn concentration in duplicates of equine plasma samples of increasing levels as measured by the Roche, Cobas-e601 hscTnT assay and the iSTAT cTnI assay. Correlation between the 2 methods was high (range: 8–3535 ng/L, r = 0.9998). Bias was evident in the Deming regression results [hscTnT = (0.6 ± 0.0028)*(cTnI*1000)–(11.5 ± 5.5)]
2. Biological variation of hscTnT levels in healthy horses
  Reference interval determination of two subset populations
    HscTnT levels were different between the Non-Competition Horses and Racing-Thoroughbreds (*p* < 0.0001) (Table 1). As such, the populations have been considered separately with respect to upper reference limits. The upper 95^th^ (90 % CI) and 99^th^ percentile of the population distribution in Non-Competitive Horses (N = 125) is 6.8 (90 % CI:6.0–12.0) and 16.2 ng/L and for Racing-Thoroughbreds (N = 178) is 14.0 (90 % CI:10.0–18.0) and 23.2 ng/L, respectively (Table 1 and Fig. 4). Lower reference limits are not clinically relevant and are defined by the LoQ of the assay (3 ng/L). 90 % CI could not be calculated for the 99^th^ percentile due N value restrictions [14]. Measureable hscTn analyte levels were obtained in 56.8 and 88.8 % for Non-Competition Horses and Racing-Thoroughbreds groups respectively.

**Fig. 4:**
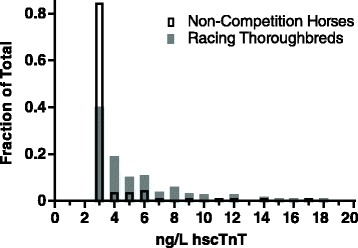
Frequency distribution for hscTnT levels in Non-Competition Horses and Racing-Thoroughbreds
  Breed, diurnal and intra-individual variation in Non-Competition Horse population
    The assay did not have sufficient sensitivity to assess between breed, diurnal effect and between-day variation in Non-Competition Horses partly due to low levels of hscTnT (43.2 % of this reference population had average values ≤ 3 ng/L) (Fig. 4).
3. Two selected case studies demonstrating clinical use of hscTnT assay
  Case one
    The 11 year old TB Chuckwagon horse had previously passed a veterinary physical and lameness examination on the morning of the race. The gelding finished the 0.5 mile race in distress and presented immediately for assessment. Examination showed cyanotic mucous membranes and persistent tachycardia (heart rate: 180 bpm). No cardiac murmurs were noted on auscultation. Ventricular tachycardia was confirmed on ECG that converted to atrial fibrillation within 30 min and spontaneously returned to sinus rhythm with supportive therapy, but no conversion treatment, within 12 h. The plasma sample taken 5 h after the race for hscTnT had a value of 221 ng/L; by 72 h the hscTnT value was 21 ng/L. Five weeks later, a standing and over-the-ground dynamic endoscopy during exercise revealed a subepiglottic cyst with a severe retroversion of the epiglottis during maximal effort.
  Case two
    The 14 year old QH mare was diagnosed with Potomac Horse Fever, and SIRS (based on tachycardia, fever, abnormal WBC count, as well as severe elevations in lactate and toxic, tacky mucous membranes) [20]. On admission the mare had severe electrolyte imbalances, endotoxemia (toxic neutrophils noted), acute renal failure, and liver impairments (Table 3). Despite emergency symptomatic treatment she remained unstable and showed deterioration in cardiovascular status (development of severe bradyarrhythmia with intermittent long sinusal pauses for up to 40 s, pale mucous membranes, and weak pulses), as well as ongoing severe diarrhea. A plasma sample taken for hscTnT 41 h following initial presentation measured 5723 ng/L. The mare was euthanized 44 h after initial presentation and post mortem findings included a moderately enlarged heart, failure of left ventricle to contract completely after rigor mortis, multifocal myocardial necrosis, and congestion of the liver all suggestive of myocardial function impairment.

**Table 3:** Case two selected laboratory results

		Time of admission	36 h Post-adm.
COMPLETE BLOOD COUNT			
	RBC (x 10^12^/L) [6.4–10.4]	10.9	5.7
	Hct (%) [30.0–47.0]	44	22.7
	WBC (x 10^12^/L) [4.9–11.1]	13.6	7.1
	NEU (x 10^12^/L) [2.5–6.9]	10.2	5.6
	LYM (x 10^12^/L) [1.5–5.1]	2.4	1.1
	MONO (x 10^12^/L) [0.2–0.6]	1.0	0.4
	EOS (x 10^12^/L) [0.0–0.8]	0	0
	BASO (x 10^12^/L) [0.0–0.1]	0.02	0.01
	PLT (K/μL) [100–250]	238	196
	Fibrinogen Manual (g/L)	7	5
CHEMISTRY			
	UREA (mmol/L) [3.6–8.9]	> 46.4	40.6
	CREA (μmol/L) [71–194]	Too high to report	Too high to report
	CA (mmol/L) [2.6–3.2]	2.1	2.7
	Total Protein (g/L) [56–79]	77	49
	ALB (g/L) [19–32]	29	18
	GLOB (g/L) [24–47]	48	31
	AST (U/L) [100–600]	410	442
	ALKP (U/L) [10–326]	195	134
	GGT (U/L) [0–87]	17	20
	TBIL (μmol/L) [0–60]	25	20
	CK (U/L) [10–350]	505	361
	LDH (U/L) [250–2070]	1694	2058
	Na (mmol/L) [133–150]	104	147
	K (mmol/L) [3.0–5.3]	2.3	2.4
	Cl (mmol/L) [97–109]	79	116
	HscTnT (ng/L)		5723
BLOOD GAS		Venous	Arterial
	pH (7.35–7.45)	7.3	7.3
	pCO_2_ (mmHg) [3.6–8.9]	40.4	38.4
	PO_2_ (mmHg)	48	61
	HCO_3_ (mmol/L) [25.0–30.0]	18	18.9
	*T* CO_2_ (mmol/L) [24–32]	19	20
	Base Excess [−5 to +5]	−9	−8
	sO_2_ (%)	77	89
	Lactate (mmol/L) [0.3–1.5]	3.0	0.9
URINE			
	USG	1.009	
	Fraction of Excretion–Na (%)		40
	Fraction of Excretion–Cl (%)		68
	Fraction of Excretion–K (%)		157

Selected laboratory results for Case two, a 14 year old QH mare diagnosed with Potomac Horse Fever, and subsequent myocardial damage

## Discussion

Adhering to the ASVCP guidelines based largely on the strict CLSI guidelines, our results indicate that the Roche Diagnostics cTnT assay (on the Cobas-e601 analyzer) can not only appropriately and adequately measure plasma concentrations of equine cTnT, but it can be classified as a’high-sensitivity’ immunoassay fit for diagnostic use in horses. Meeting the CLSI guidelines, our study defined the reference intervals and determined the upper 95^th^ (90 % CI) and 99^th^ percentile of the population distribution in Non-Competitive Horses (N = 125) is 6.8 (90 % CI: 6.0–12.0) and 16.2 ng/L and for Racing-Thoroughbreds (N = 178) is 14.0 (90 % CI: 10.0–18.0) and 23.2 ng/L, respectively (Table 1, Fig. 4). This study provides additional information key to interpreting hscTnT results in clinical context as compared with recent reports [6, 7].

The epitopes detected by this hscTnT assay were conserved between humans and horses with 100 % homology detected when comparing human adult cTnTand equine cTnT (Fig. 1). Utilizing a series of validation experiments according to industry guidelines, the assay was challenged with detecting and measuring equine cTnT [15, 16]. Homogenates of equine cardiac and skeletal muscle allowed for the determination of specificity of the assay for hscTnT in cardiac versus skeletal muscle. Cross-reactivity with the skeletal muscle isoform of Troponin-T was minimal, indicating excellent assay specificity for the cardiac isoform. Purified equine cardiac troponin obtained from harvested equine myocardium allowed for the determination of linearity explored to levels exceeding the expected upper limit of the assay according to ASVCP guidelines (up to 3526.7 ng/L) [16]. This method differed from that used in the Van Der Vekens study [6] (in which diluted pooled samples from clinical cases were used to assess linearity up to 1098. 8 ng/L) and allowed assessment of assay linearity at higher analyte levels. Linearity of the assay in our study was assessed by linear and polynomial regression of the data and difference plot with Total Error criteria, whereas the Van Der Vekens group used simple linear regression analysis. Our work demonstrated good assay linearity over the low range of cTnT concentrations, but slightly over-recovers at high levels. However, 391 ng/L is > 10 times our defined URLs, so linearity to this level should confer good diagnostic sensitivity for distinguishing sick from healthy populations. For an accurate quantitative determination for clinical purposes it is therefore recommended to dilute specimens > 391 ng/L into the linear range for future studies. No such dilutions were performed on any clinical samples utilized throughout this study.

The within-run and between-run precision values were similar to those reported for measurement of human cTnT and were within CLSI standards [21]. In humans this assay has complied with the standards set forth by the International Federation of Clinical Chemistry and Laboratory Medicine to be defined as a’high-sensitivity’ assay [4]. Based on the same stringent criteria, our study shows that this assay can be classified as a ‘high-sensitivity’ assay appropriate for diagnostic use in horses. Specifically we showed that the total imprecision was ≤ 10 % at the 99^th^ percentiles for our two reference populations (16.22 and 23.21 ng/L), and measureable cTn analyte levels were obtained in 56.8 and 88.8 % for Non-Competition Horses and Racing-Thoroughbreds groups respectively.

It is critical for clinicians to understand certain basic characteristics of assays, development of reference intervals, and establishment of decision thresholds in order to critically interpret troponin assay use reported in the equine literature, and to successfully apply the assays available for use in practice. The limit of quantitation (LoQ) is the concentration above which quantitative results may be obtained with a specific degree of confidence (ie.CV ≤ 10 %), and according to the CLSI guidelines is recommended over the limit of detection (LoD) for setting the lower reporting limits of a test for clinical use. Importantly, only concentrations exceeding an assay’s LoQ should be reported as a numerical value. The LoQ can, for example, be significantly greater than the LoD, as is reported in human plasma (LoD =3 ng/L, but LoQ = 13.5 ng/L) [21]. All equine studies to date using cardiac troponins mainly report values above manufacturers’ defined LoDs, without verifying [6, 7, 9, 10], or adhering to a determined functional sensitivity (LoQ) [8]. The LoQ that we determined for equine plasma using Roche’s hscTnT assay was validated at 3 ng/L (10 % CV). It is possible that the true LoQ may be at a level lower than 3 ng/L, but because the manufacturer limits all reported values to 3 ng/L, lower levels could not be validated. Additionally, the 10 % CV at 3 ng/L which is lower than what was observed in within-run precision Level 1 (14.9 % at 6.5 ng/L) may indicate that at 3 ng/L the assay is approaching non-linear dose response. Nevertheless, our results imply that any Roche hscTnT assay value reported by the e601-Cobas instrument is reliable for use in horse plasma.

The hscTnT assay reported values < 3 ng/L in 43.2 and 11.2 % of the Non-Competition Horses and Racing-Thoroughbreds, respectively, which is similar to the validation study in heathy humans (20.1 % at < 3 ng/L) [21]. This limited the determination of biological variation, measured as intra-individual, inter-individual variation (between-breed), reference change values (RCVs), and the index of individuality values. The ability of this assay to function at these low concentrations (LoQ level) is unique when compared to the only cTnI assay similarly validated appropriately by ASVCP and CLSI standards for use in horses [8], and thus continues to show promise for its’ ability to detect myocardial dysfunction as well as the response of equine myocardium to various exercise regimes. Comparison of hscTnT analyte levels (measured on the Roche Diagnostics hscTnT assay) and cTnI analyte levels (measured on the the iSTAT^4^) in purified equine cardiac troponin samples, using a Deming regression, showed excellent correlation. Of note, a Blant-Altman graph represents a bias plot and was not used to compare the two assays as the numerical value of any bias between hsTnT and cTnI is not clinically interpretable when comparing the relative recoveries of these two entirely different molecules. As human laboratories trend toward only using’high-sensitivity’ troponin assays, the manufacturing of older generation assays may discontinue.

While troponin assays’ use continues to grow in veterinary fields clinicians must be aware that “all troponin assays are NOT created equal” [22]. In the thorough review by Rossi et al. the use of troponin I assays in horses, as well as their associated limitations and challenges, are highlighted [5]. The analysis and standardization for cTnT is beneficially more straightforward than for cTnI. All assays for cTnT are marketed by one manufacturer^1^, and instruments are calibrated to the same reference material. It has been demonstrated that Roche’s cTnT immunoassay detects binary (cTnT-I and cTnT-C), ternary complexes (cTnT-I-C) of troponin, and the free-cTnT form, as well as capably measures cTnT degradation by-products and phosphorylated-cTnT in circulation [23]. Therefore, cTnT degradation does not affect harmonization between different assays as previously reported for the cTnI assays [24]. The effect of EDTA in the cTnT assay seems to be minimal, whereas differences between serum and plasma values have been reported for a number of cTnI methods [4]. In human patient samples, cTnT appears to be very stable at room temperature, 4 °C, and when repeatedly frozen and thawed [21]. Similarly our study demonstrated excellent stability of equine samples frozen, stored, and then thawed over various time periods.

A goal for any assay is to establish decision thresholds to distinguish among different groups. In human medicine, an algorithm has been agreed upon to differentiate healthy individuals from those suffering an AMI that includes the use of cTn assays but importantly includes other criteria such as presence of clinical signs, and other diagnostic imaging findings. Furthermore it recommends obtaining individuals’ cTn levels at a second time point to increase the specificity of the assay and promotes the use a delta cut-off instead of single diagnostic cut-off value [25]. Other factors, such as exercise exposure, must be considered when interpreting the results of individual hscTnT analyte levels. This will be particularly relevant when this assay is to be used on performance horses, and thereby warrants further study into the kinetics of hscTnT in the exercising horse.

Utilizing the ROC curve method, a calculated hscTnT 6.6 ng/L cut-off point for distinguishing healthy horses from those with primary myocardial disease has been recently published using the same Roche hscTnT assay [7]. Applying this cut-off point to our two defined reference populations would imply 5 % of the Non-Competition, and 22 % of the Racing-Thoroughbreds would be classified as having primary myocardial disease. This interpretation is unlikely, and indicates caution must be used when applying this cut-off point to various horse populations. The discrepancy may be partly explained by the inclusion of only 20 healthy horses in the reference population described by Van Der Vekens et al. [7], as compared to our N = 125 Non-Competition and N = 178 Racing-Thoroughbred reference populations. Including a minimum of 120 horses in each of our groups provided populations for which reference intervals could be defined by statistical methods in compliance with ASCVP guidelines and provide better whole population normal hscTnT level estimations. The use of only 20 horses in the reference population used by Van Der Vekens et. al. [7] led to a “tight” reference range, and significant overlap between their normal group and those classified with primary myocardial disease. Their low cut-off point was necessary to maintain acceptable sensitivity of the assay, enabling it to distinguish between their two groups, but is at the expense of possibly misclassifying an unacceptably high number of healthy horses as diseased. Their healthy horse group is most likely more similar to our Non-Competition group of horses (than our Racing-Thoroughbred group), but the authors did not describe the use or fitness status of the members of this group in which 10/35 were Trotters that may have been in training or racing. Regardless, a hscTnT 6.6 ng/L cut-off point differentiating healthy from those with primary myocardial is likely more appropriately applied to relatively sedentary horses rather than racing populations. Future studies may be warranted to determine cardiac troponin cut-off points specific to racing horses.

The inclusion of only two case studies in our study is few, but nevertheless demonstrate applications of the hscTnT assay unique to those recently presented [7]. In case one we report an adult Thoroughbred racing gelding that presented for near collapse event, distress, clinical signs of hypoxia (cyanosis), cardiac abnormalities identified on auscultation and ECG, and subsequently abnormally high levels of hscTnT. The later over-the-ground dynamic endoscopic confirmation of severe epiglottic retroversion with a subepiglottic cyst and dorsal displacement of the soft palate suggests a possible association between the clinical presentation of impaired gas exchange and cardiac function as documented by elevations in the myocardial biomarker hscTnT. We do however acknowledge that a cardiac ultrasonographic assessment is missing, which limited the full characterization of his cardiac status and the ability to determine the complete etiology of his presentation. The elevated value detected 72 h later demonstrates persistence of the biomarker cTnT further emphasizing its clinical utility, as the practitioner can take samples at time points slightly past onset of acute condition without missing the opportunity to detect myocardial insult. Case two demonstrates a severe, multi-organ system dysfunction likely related to SIRS and endotoxemia associated with Potomac horse fever. Increases in cardiac troponin have been previously reported in experimentally-induced endotoxemia [9]. The diagnosis of myocardial function impairment in this case was based on clinical presentation, ECG monitoring, hscTnT levels, and post-mortem evaluation but may have been strengthened by completion of cardiac ultrasonography. Case two exemplifies the level to which hscTnT may be seen clinically (5723 ng/L in this case as compared to the highest level of 1341 ng/L recorded by the Van de Vekens group in a horse with primary myocardial disease) [7]. Despite the fact that our linearity experiment showed the assay has slight over-recovery of the hscTnT analyte > 391 ng/L value, tracking the trend over time in severely elevated values in cases such as this would be of interest to the clinician with respect to case management and prognosis [11].

Additional limitations of our study include the abbreviated resting-ECG (2 min) on all horses included in the two reference populations, as well as the absence of cardiac ultrasonography. These methods reflect field-study condition constraints. More thorough screening may have further identified individuals that were inappropriate for study inclusion as “healthy horses”. Our total samples numbers included, and the absence of significant outliers suggest however that the reference populations appropriately reflect more generalized healthy populations.

## Conclusions

In conclusion, this report has validated, according to the rigorous recommendations of the CLSI, the only’high-sensitivity’ troponin assay (Roche hscTnT, Cobas-e601) for use in horses. The current literature is lacking many of the validation experiments conducted in our study, and thus the data published here significantly adds to the conclusions of Van der Vekens et al. [6, 20]. In our study reference intervals for the hscTn-T assay in healthy adult Non-Competition and Racing Thoroughbred horses were established, as were assay performance characteristics, and sample collection protocols in equine plasma samples. While the assay remains insufficiently sensitive to provide biological variation information in mainly sedentary healthy horses at this time, it will prove extremely useful for assessment of subtle myocardial injury as well as the effects of exercise on equine myocardium.

## Abbreviations

AMI, acute myocardial injury; ASVCP, American Society of Veterinary Clinical Pathology; Bpm, beats per minute; CI, confidence interval; CLSI, Clinical and Laboratory Standards Institute; cTn-I, cardiac Troponin-I; cTnT, cardiac Troponin-T; CV, coefficient of variation; ECG, electrocardiogram; EDTA, Ethylenediaminetetraacetic acid; hscTnT, high-sensitivity cardiac Troponin-T; L1, level 1; L2, level 2; L3, level 3; LoD, limit of detection; LoQ, limit of quantitation; NACB, National Academy of Clinical Biochemistry; PCR, polymerase chain reaction; RCVs, reference change values; ROC, receiver operating characteristic; SIRS, systemic inflammatory response syndrome; URL, upper reference limit; WBC, white blood cell.

## Footnotes

2 Cobas-e601, Roche Diagnostics, Indianapolis, IN.

3 Elecsys diluent multi-assay, Roche Diagnostics, Indianapolis, IN.

5 Televet-100, Engel Engineering, Offenbach, Germany.

4 iSTAT-2, Heska Corp, Loveland, CO.

1 Troponin-T hs, Roche Diagnostics, Indianapolis, IN.
